# Neonatal heavy metals levels are associated with the severity of neonatal respiratory distress syndrome: a case–control study

**DOI:** 10.1186/s12887-022-03685-5

**Published:** 2022-11-04

**Authors:** Khalid M. Mohany, Osama Mahmoud El-Asheer, Yaser F. Abdel Raheem, Ahmed Abd-Elrasoul sayed, Mona Abd El-Hamid Hassan El-Baz

**Affiliations:** 1 Department of Medical Biochemistry and Molecular Biology, Faculty of Medicine, Assiut University, Assiut, Egypt; 2 Department of Medical Biochemistry and Molecular Biology, Faculty of Medicine, Assiut University, EL Gammaa Street, Assiut city, 00201146007069 Egypt; 3 Department of Pediatrics, Faculty of Medicine, Assiut University, Assiut, Egypt; 4 Clinical Pharmacist at Assiut University Children Hospital, Assiut, Egypt

**Keywords:** Heavy metals, SP-D, CTnI, Hs-CRP, NRDS

## Abstract

**Background:**

This case–control study aimed to compare lead (Pb), cadmium (Cd), and arsenic (As) levels in neonates with respiratory distress syndrome (NRDS) with those levels in normal neonates and tested their associations with the severity of NRDS indicated by the levels of serum surfactant protein D (SP-D) and cord blood cardiac troponin I (CTnI), and high-sensitive C-reactive protein (hs-CRP).

**Methods:**

The study included two groups: G1 (60 healthy neonates) and G2 (100 cases with NRDS). Cord blood Pb, erythrocytic Cd (E-Cd), neonatal scalp hair As (N-As), maternal urinary Cd (U-Cd), and arsenic (U-As) were measured by a Thermo Scientific iCAP 6200, while CTnI, hs-CRP, and SP-D by their corresponding ELISA kits.

**Results:**

The levels of cord blood Pb, E-Cd, N-As, U-Cd, U-As, SP-D, CTnI, and hs-CRP were significantly higher in G2 than G1 (*p* = 0.019, 0.040, 0.003, 0.010, 0.011, < 0.001, 0.004, < 0.001, respectively). While the birth weight, and APGAR score at 1, 5 and 10 min were significantly lower in G2 than G1 (*p* = 0.002, < 0.001, < 0.001, < 0.001, respectively). The levels of the studied heavy metals correlated positively with the levels of SP-D, CTnI, and hs-CRP.

**Conclusion:**

Heavy metals toxicity may be accused to be one of the causes of NRDS especially if other apparent causes are not there. Measuring and follow-up of heavy metal levels should be considered during pregnancy.

## Background

Neonatal respiratory distress syndrome (NRDS) is a significant cause of newborn mortality that is characterized by lung immaturity and a deficiency in lung surfactant [1]. It affects mainly preterm neonates but also term infants may be affected [2].

The lung surfactant is mostly lipid-dense, consisting mainly of phospholipids, with smaller percentages of neutral lipids and proteins [3]. Surfactant proteins (SPs), members of the collectin group of defense lectins, are important for optimal respiratory function. There are four types of SPs namely A, B, C, and D. The SP-A and SP-D regulate inflammatory processes in the lung [3]. The assessment of serum SP-D levels in NRDS is employed as a biomarker of the severity and prognosis of unfavorable outcomes [4].

Exposure to heavy metals is inevitable as they are widely used in industry, households, agriculture, cosmetics, and medical preparations [5]. The lung is very vulnerable to these environmental toxins during development, both in utero and early postnatal [6, 7]. These heavy metals are usually present in airborne particulate matter (PM) [8]. Inhaled PM interacts with the lung surfactant located in the alveolar lining causing different pulmonary diseases. The inverse effect of PM is proportionate to the solubility of heavy metals in the pulmonary fluid [9]. Of these heavy metals, lead (Pb), cadmium (Cd), and arsenic (As) are widely studied and known to induce immunological changes and have been linked to premature labor [10, 11].

The current study aimed to compare the levels of cord blood Pb, cord erythrocytic Cd (E-Cd), and neonatal scalp hair As (N-As) in neonates with NRDS with those levels in normal neonates. Also, maternal urinary Cd (U-Cd) and urinary arsenic levels (U-As) were measured and compared in both groups. In addition, the study tested the associations of these levels with the severity of NRDS indicated by the levels of serum SP-D and cord blood the stress biomarkers; cardiac troponin I (CTnI) and high-sensitive C-reactive protein (hs-CRP).

## Methods

The current work is a case–control study that was conducted between August 2021 and April 2022 in the medical biochemistry department, Assiut University, in cooperation with the Assiut university children’s hospital and with the University Woman hospital. The study included 160 neonates divided into two groups; G1 (the control group; 60 healthy neonates who accepted to participate) and G2 (cases; 100 consecutive cases with NRDS who were admitted to the neonatal intensive care unit in Assiut University Children’s Hospital. The mothers of these neonates were admitted to Assiut University Woman hospital for delivery). After excluding babies with the exclusion criteria mentioned below, the diagnosis of the NRDS was depending on the clinical manifestations (disturbed respiration, grunting, intercostal retractions, and cyanosis), laboratory analysis (low blood oxygen (hypoxia), elevated arterial CO_2_ (hypercapnia), and acidosis, and the radiological findings [12, 13].

### Exclusion criteria

The neonate was excluded from the study when exhibited congenital anomaly, infections, or inflammation, any other systemic diseases, or signs of hypoxic-ischemic encephalopathy (178 infants were excluded according to these criteria).

### Medical histories and physical examinations

Complete medical and obstetric histories were taken from the mothers. For neonates, full medical examination, anthropometric measurements, and APGAR scores at 1, 5, and 10 min after birth were done.

### Sampling and laboratory analysis

#### Analysis of maternal urinary Cd (U-Cd) and arsenic (U-As)

Five ml of freshly voided midstream urine samples (or by catheter) were collected aseptically. The samples were centrifuged to get rid of sediments then a drop of HCl (conc.) was added to stop any bacterial growth. Then the samples were kept at -70 °C till the measurement the U-Cd and U-As levels by an inductively coupled plasma-optical emission spectrophotometer (Thermo Scientific iCAP 6200) [14]. The alkaline picrate method was used to measure urinary creatinine [15]. The maternal urinary Cd levels were expressed as (μg/g creatinine) while urinary As levels were expressed as (μg/l) [16].

#### Analysis of cord blood CTnI, hs-CRP, Pb (Pb) and E-Cd

The umbilical cord was clamped immediately after delivery, adequately cleaned with an antiseptic agent, and cut. Six ml of cord arterial blood was withdrawn. Two ml of them were left to clot for 20 min, centrifuged, and the serum was separated and kept at -70 °C till the measurement CTnI by the Dimension® (RXL-Max/Xpand) clinical chemistry system with the electrochemiluminescence immunoassay kit (Cat. No: RF421C) and hs-CRP by the ADVIA Chemistry using the kit (Cat.NO:05006455). Both kits were supplied by Siemens Healthcare Diagnostics Products GmbH, Marburg, Hessen, Germany.

The remaining 4 ml were divided between two EDTA-containing tubes; Two ml were kept as whole blood and the other 2 ml as RBCs concentrate (after centrifugation at 4000 rpm for 15 min and washing 3 occasions with normal saline). The whole blood and RBCs were digested by the method described by Marouf (2011) [17]. Briefly, the samples were diluted 1:2 with 20% trichloroacetic acid solution and heated in an oven at 90 Ċ for 15 min. After cooling and centrifugation (3000 rpm for 15 min), the supernatants were collected and their Pb and Cd contents were determined by a Thermo Scientific iCAP 6200 [14, 17].

#### Analysis of the neonatal scalp hair As (N-As)

Stainless steel scissors were used to cut hair samples from the back of the newborn’s head and collected into a labelled polythene bag, sealed with a zipper. They were cut into small pieces, cleansed, and rinsed five times with deionized water. Washed samples were oven-dried for 24 h at 60 °C before being weighed. For digestion, 2 ml of HNO_3_ (65%) was applied to 0.1 g of hair samples in graduated polypropylene tubes for 24 h. Half ml of H_2_O_2_ (30%) was added to the tubes. The final digested samples were diluted to 10 ml by ultrapure deionized water. After cooling to room temperature, the samples were ready for the measurement of N-As by Thermo Scientific iCAP 6200 [14, 18].

#### Analysis of neonatal serum SP-D levels

Two ml of venous blood were obtained from each neonate, left to clot for 20 min and centrifuged at 3000 rpm for 15 min. The sera were separated and collected into labeled Eppendorf tubes and frozen at -70 °C. The level of SP-D was measured using human SP-D ELISA kit (Thermo-Fisher Scientific, USA, Cat. No: EH436RB), after the manufacturer’s instructions.

### Statistical analysis

The collected data were examined by SPSS (v.26). After testing the data normality, Mann Whitney U and Chi-square test were used to compare the quantitative and qualitative variables in the two groups [19]. The correlations between the studied continuous variables were tested by Spearman coefficient (*rho*) [20]. The receiver operating characteristic curve (ROC) was performed to assess the variables’ ability to differentiate neonates with NRDS from healthy neonates [21]. *p*-Value ≤ 0.05 was adjudged to be statistically significant [19].

## Results

### Sociodemographic data, anthropometric measurements, APGAR score, and other studied biomarkers in the G1 and G2 (Table 1)

**Table 1:** Sociodemographic data, anthropometric measurements, APGAR score, and other studied biomarkers levels in the G1 and G2

		**G1 (control)** **(** ***N*** ** = 60)**	**G2 (NRDS)** **(** ***N*** ** = 100)**	***p*** **.Value**
		Mean ± SD	Mean ± SD	
**Gestational age at birth**	**Preterm**	12 (20%)	70 (70%)	< 0.001*
	**Full-term**	48 (80%)	30 (30%)	
**Maternal exposure to smoking**	**Passive smokers**	25 (41.67%)	75 (75%)	< 0.001*
	**Non-smokers**	35 (58.33%)	25 (25%)	
**Maternal exposure to insecticides, pesticides..etc**	**Yes**	22 (36.66%)	68 (68%)	< 0.001*
	**No**	38 (63.33%)	32 (32%)	
**Residence area**	**Urban**	19 (31.67%)	44 (44%)	0.262*
	**Semiurban**	19 (31.67%)	29 (29%)	
	**Rural**	22 (36.67%)	27 (27%)	
**Neonatal gender**	**Male**	19 (31.67%)	33 (33%)	0.862*
	**Female**	41 (68.33%)	67 (67%)	
**Birth weight (gm)**		2650 ± 749	1788 ± 814	< 0.001
**APGAR score**	**at 1 min**	6.42 ± 1.19	4.44 ± 1.39	< 0.001
	**at 5 min**	8.35 ± 0.92	6.99 ± 1.14	< 0.001
	**at 10 min**	9.28 ± 0.59	8.78 ± 0.85	< 0.001
**Hemoglobin (g/dl)**		12.21 ± 2.31	13.61 ± 2.17	< 0.001
**Hematocrit value**		35.94 ± 10.02	43.49 ± 9.07	< 0.001
**Pb (μg/dl)**		26.3 ± 12.17	31.12 ± 12.58	0.019
**E-Cd (μg/dl)**		2.49 ± 1.79	3.12 ± 1.98	0.04
**N-As (μg/g dry hair)**		5.78 ± 4.31	7.82 ± 4.17	0.003
**U-Cd (μg/g creatinine)**		18.52 ± 8.32	25.57 ± 19.79	0.010
**U-As (μg/l)**		202.97 ± 84.20	246.16 ± 112.99	0.011
**SP-D (ng/ml)**		7.48 ± 3.54	9.87 ± 3.43	< 0.001
**CTnI (ng/ml)**		0.043 ± 0.048	0.109 ± 0.172	0.004
**hs-CRP (mg/l)**		0.434 ± 1.544	2.918 ± 1.884	< 0.001

Mann Whitney U test was used to compare continuous variables

*NRDS* Neonatal respiratory distress syndrome, *SD* Standard deviation, *APGAR* Appearance, *Pulse* Grimace, Activity, and Respiration, *RDS* Respiratory distress syndrome, *Pb* Cord blood lead, *E-Cd* cord erythrocytic cadmium, *N-As* Neonatal scalp hair arsenic, *U-Cd* Maternal urinary cadmium, *U-As* Maternal urinary arsenic, *SP-D* Surfactant protein D, *CTnI* Cardiac troponin I, *hs-CRP* High-sensitive C-reactive protein

^*^Chi-square test was used

The levels of cord CTnI, hs-CRP, cord blood Pb, E-Cd, N-As, SP-D, U-Cd, and U-As were significantly higher in neonates suffering from NRDS (G2) than the healthy control (G1). While the birth weight, and APGAR score at 1, 5 and 10 min were significantly lower in G2 than G1.

The percentages of preterm infants, exposure to passive smoking, insecticides, pesticides, and other possible sources of heavy metals were higher in G2 than G1. Non-significant differences were found regarding maternal residence and neonatal gender between the two groups (Table 1). Also, non-significant difference was found between the two groups regarding the percentages of working and non-working mothers (45% and 55% in G1 and G2 = 54% and 46% in G2, respectively and *p* = 0.27).

A significant lower percentage of mothers who had a history of using antenatal steroids was found in G2 (17%) than those in G1 (70%) (*p* < 0.001) (data not shown in table).

### Correlations (Tables 2 & 3, Figs. 1, 2 & 3)

**Table 2:** Correlations of the heavy metals’ levels with the levels of SP-D, CTnI and hs-CRP in the whole study sample (*N* = 160)

		**Pb(μg/dl)**	**E-Cd(μg/dl)**	**N-As (μg/g dry hair)**	**U-Cd (μg/g creatinine)**	**U-As(μg/l)**
**SP-D (ng/ml)**	*rho*	0.167	0.062	0.268	0.176	0.329
	p	0.035	0.434	0.001	0.026	< 0.001
**CTnI(ng/ml)**	*rho*	0.416	0.193	0.069	0.091	-0.064
	p	< 0.001	0.014	0.383	0.253	0.421
**hs-CRP (mg/l)**	*rho*	0.184	0.163	0.160	0.133	0.215
	p	0.02	0.04	0.044	0.092	0.006

*SP-D* Surfactant protein D, *CTnI* Cardiac troponin I, *hs-CRP* High-sensitive C-reactive protein, *Pb* Cord blood lead, *E-Cd* Cord erythrocytic cadmium, *N-As* Neonatal scalp hair arsenic, *U-Cd* Maternal urinary cadmium, *U-As* Maternal urinary arsenic, *rho* Spearman correlation, p: *p*-Value

**Table 3:** Correlations of the heavy metals’ levels with the levels of SP-D, CTnI and hs-CRP in G1 and G2

		**Pb(μg/dl)**	**E-Cd(μg/dl)**	**N-As (μg/g dry hair)**	**U-Cd (μg/g creatinine)**	**U-As(μg/l)**
**G1 (Control, ** ***N*** ** = 60)**						
**SP-D (ng/ml)**	*rho*	-0.221	-0.088	0.188	0.109	0.069
	P	0.090	0.502	0.15	0.409	0.600
**CTnI(ng/ml)**	*rho*	-0.228	-0.128	-0.045	-0.056	-0.166
	P	0.08	0.329	0.734	0.672	0.206
**hs-CRP (mg/l)**	*rho*	-0.109	0.075	-0.026	0.124	-0.041
	P	0.407	0.571	0.844	0.346	0.755
**G2 (NRDS, ** ***N*** ** = 100)**						
**SP-D (ng/ml)**	*rho*	0.316	0.069	0.226	0.133	0.389
	P	0.001	0.496	0.024	0.188	< 0.001
**CTnI(ng/ml)**	*rho*	0.535	0.223	0.032	0.055	-0.114
	P	< 0.001	0.026	0.751	0.59	0.257
**hs-CRP (mg/l)**	*rho*	0.196	0.096	0.069	0.002	0.188
	P	0.05	0.341	0.498	0.981	0.062

*SP-D* Surfactant protein D, *CTnI* Cardiac troponin I, *hs-CRP* High-sensitive C-reactive protein, *Pb* Cord blood lead, *E-Cd* Cord erythrocytic cadmium, *N-As* Neonatal scalp hair arsenic, *U-Cd* Maternal urinary cadmium, *U-As* Maternal urinary arsenic, *rho* Spearman correlation (rho), *p p*-Value, *NRDS* Neonatal respiratory distress syndrome

**Fig. 1:**
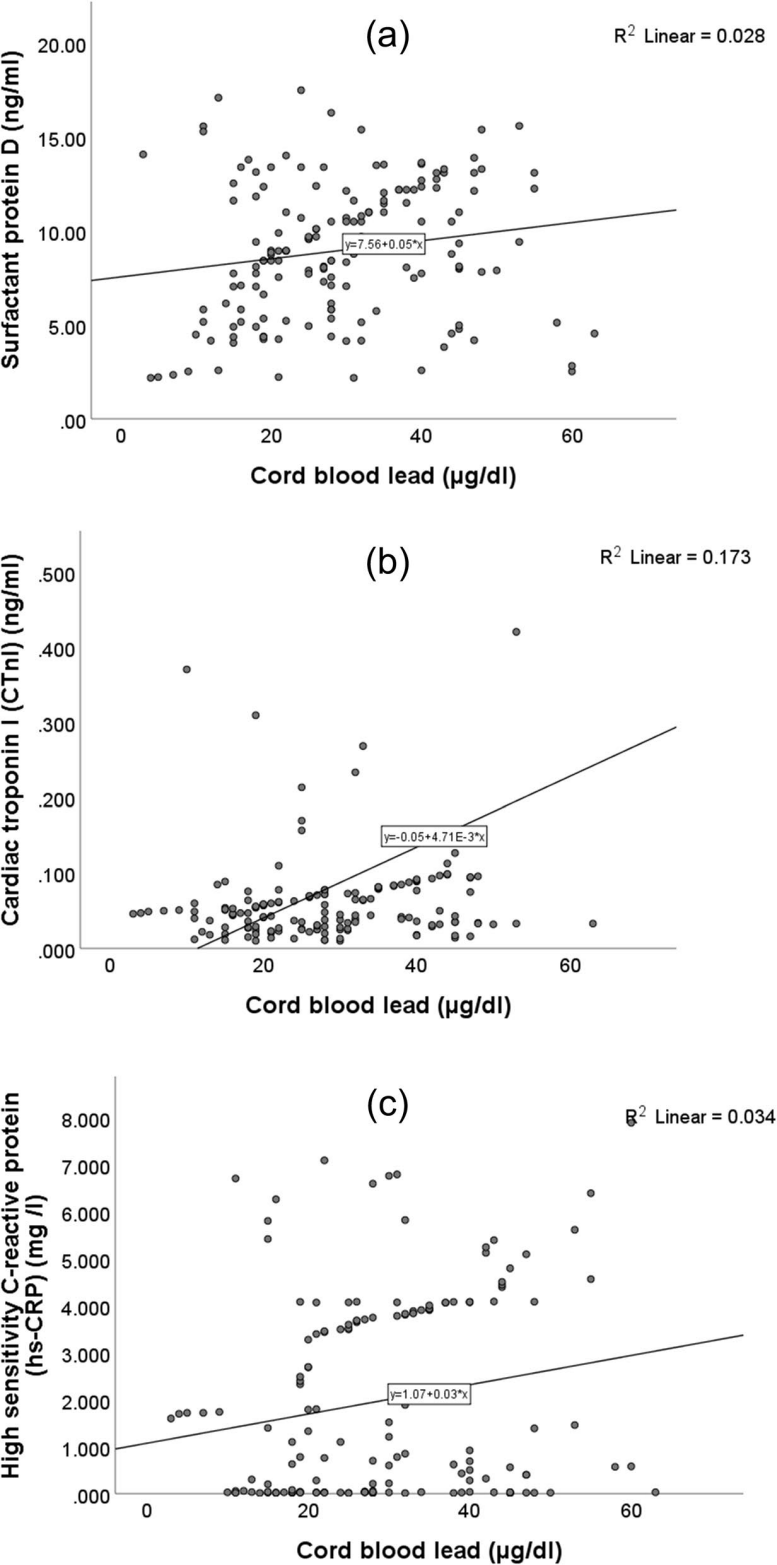
Correlations of cord blood lead levels with the levels of (**a**) surfactant protein D, (**b**) cardiac troponin I, and (**c**) high-sensitivity C-reactive protein in the whole study sample

**Fig. 2:**
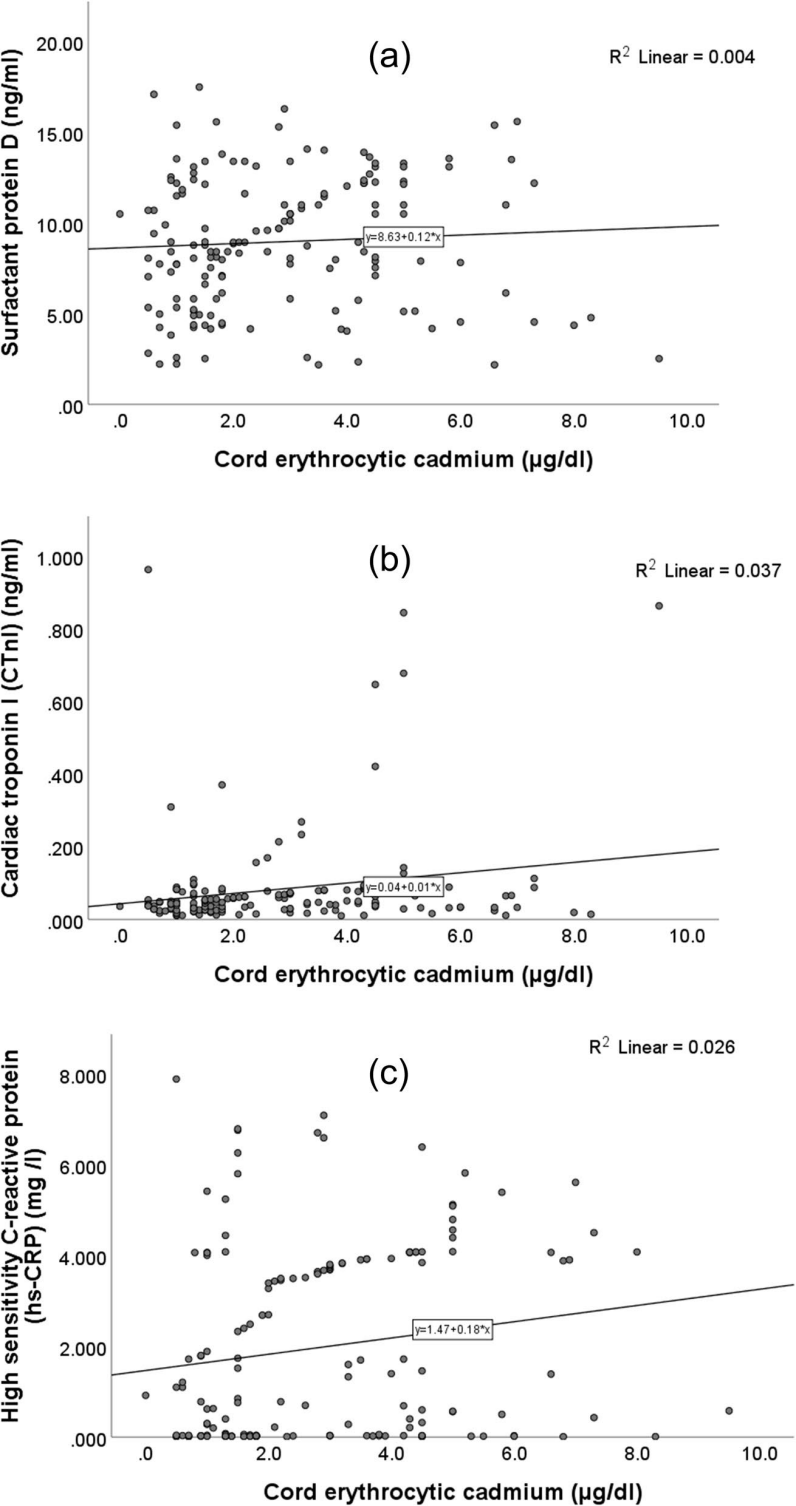
Correlations of cord erythrocytic cadmium levels with the levels of (**a**) surfactant protein D, (**b**) cardiac troponin I, and (**c**) high-sensitivity C-reactive protein in the whole study sample

**Fig. 3:**
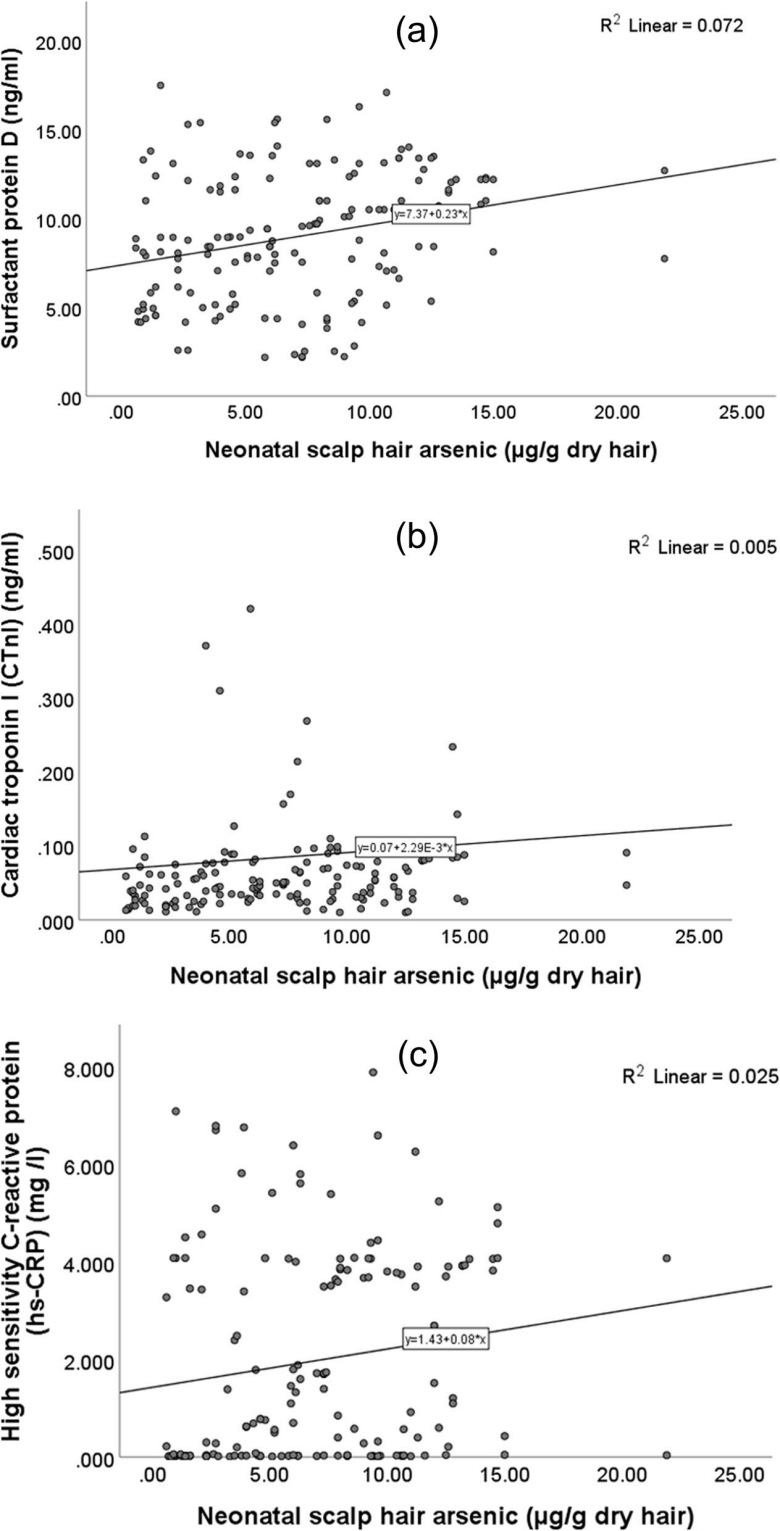
Correlations of neonatal scalp hair arsenic levels with the levels of (**a**) surfactant protein D, (**b**) cardiac troponin I, and (**c**) high-sensitivity C-reactive protein in the whole study sample

The cord E-Cd levels correlated positively with the maternal U-Cd levels (*r* = 0.232, *p* = 0.003) and the N-As levels correlated positively with the maternal U-As (*r* = 0.466, *p* < 0.001) (data not shown in the tables).

Cord blood Pb levels correlated positively with the levels SP-D, CTnI, and hs-CRP in whole study sample and in G2. Cord E-Cd levels correlated with the levels of CTnI and hs-CRP in whole study sample and only with the levels of CTnI in G2. The N-As and the maternal U-As levels correlated positively with the levels of SP-D and hs-CRP in the whole study sample but only with the levels of SP-D in G2. Maternal U-Cd levels correlated positively with the levels of SP-D in the whole sample. The levels of SP-D were correlated positively with the levels of hs-CRP in the whole sample (*r* = 0.338, *p* < 0.001) (data not shown in the tables).

Also, in the whole study sample, Cord blood Pb levels correlated negatively with the birth weights, and APGAR score at 1 and 5 min (*r* = -0.368, -0.268, -0.171, and -0.224, *p* =  < 0.001, 0.001, 0.031, and 0.004, respectively). Cord E-Cd correlated negatively with the birth weights, and APGAR score at 1 and 5 min (*r* = -0.352, -0.227, and—0.195, *p* =  < 0.001, 0.004, and 0.013 respectively). The levels of N-As correlated negatively with the birth weights, and APGAR score at 1 and 5 min (*r* = -0.294, -0.250, and -0.170, *p* =  < 0.001, 0.001, and 0.032 respectively). Maternal U-Cd correlated negatively with APGAR score at 10 min (*r* = -0.169, *p* = 0.032). Maternal U-As correlated negatively with birth weights, and APGAR score at 1 and 5 min (*r* = -0.420, -0.287, and -0.173, *p* =  < 0.001, < 0.001, and 0.028 respectively). SP-D correlated negatively with birth weights and APGAR score at 1 min (*r* = -0.344, and -0.342, *p* < 0.001, and < 0.001 respectively). The levels of CTnI correlated negatively with birth weights, and APGAR score at 1 and 5 min (*r* = -0.314, -0.301, and -0.253, *p* =  < 0.001, < 0.001, and 0.001 respectively). The levels of hs-CRP correlated negatively with birth weights, and APGAR score at 1 and 5 min (*r* = -0.403, 0.413, and -0.377, *p* =  < 0.001, < 0.001, < 0.001 respectively).

### ROC analysis

**Fig. 4:**
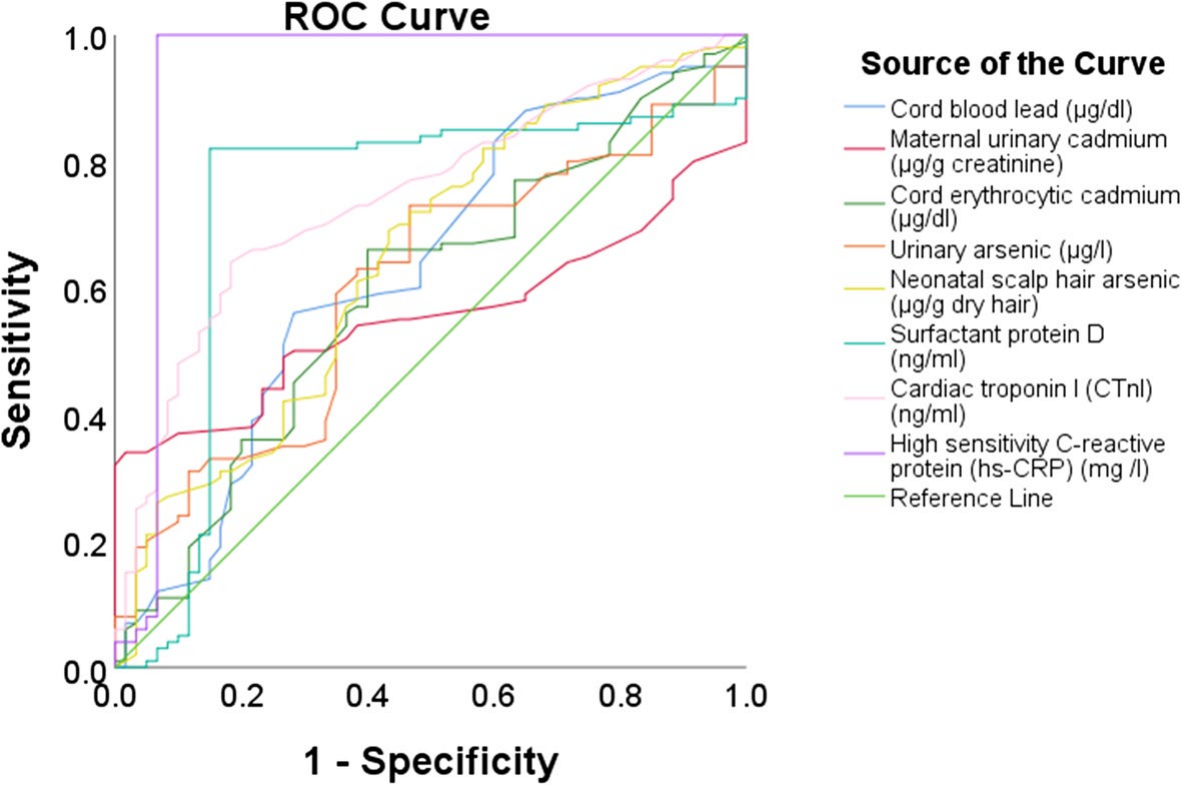
The efficacy of cord blood Pb, maternal urinary Cd, cord erythrocytic Cd, N-As, SP-D, maternal urinary As, surfactant protein D, cardiac troponin I (CTnI), and high sensitive C-reactive protein (hs-CRP) in distinguishing healthy neonates from those suffering from neonatal respiratory distress syndrome (NRDS)

The efficacy of cord blood Pb, E-Cd, N-As, maternal U-Cd, U-As, SP-D, CTnI, and hs-CRP in distinguishing healthy neonates from those with NRDS, their AUCs, Cut-off points, sensitivities, and specificities are shown in Fig. 4 and Table 4.

**Table 4:** Roc analysis for the studied variables and their ability to differentiate neonates with NRDS from healthy neonates

**Item**	**AUC**	**Cut-off point**	**Sensitivity (%)**	**Specificity (%)**
PB (μg/dl)	0.627	30.5	51	26
E-Cd (μg/dl)	0.592	3.32	43	28
N-As (μg/g dry hair)	0.647	7.35	53	35
U-Cd (μg/g creatinine)	0.556	25.5	44	26
U-As (μg/l)	0.603	213	52	35
SP-D (ng/ml)	0.728	8.82	72	15
CTnI (ng/ml)	0.745	0.054	59	17
hs-CRP (mg/l)	0.937	29	56	28

*NRDS* Neonatal respiratory distress syndrome, *Pb* Cord blood lead, *E-Cd* Cord erythrocytic cadmium *N-As* Neonatal scalp hair arsenic, *U-Cd* Maternal urinary cadmium, *U-As* Maternal urinary arsenic, *SP-D* Surfactant protein D, *CTnI* Cardiac troponin I, *hs-CRP* High-sensitive C-reactive protein

## Discussion

The NRDS because of inadequate lung surfactant is a major cause of morbidity in preterm infants, especially during the 1^st^ month of life [22]. In the current work, the severity and occurrence of NRDS are inversely related to the gestation age (GA) with severe manifestations noticed in the low-weight premature neonates. This finding is in accordance with the results of many previous studies. Yadav et al. (2020) reported that full term newborn are less frequently affected with NRDS than preterm newborn [23]. Also, Donda et al. (2019) reported that 40–50% of neonates born < 32 weeks of gestation developed NRDS and this percentage increased up to 90% in neonates born at less than 28 weeks of gestation [24].

Pregnant women's exposure to heavy metals, such as Pb, Cd, and As, is common as they are widely used in industry, households, agriculture, cosmetics, and medicine [5]. Lead has widespread sources including disintegrating lead paints, gasoline exhaust, insecticides, cigarettes, newspapers, and Kohl fixtures [5]. Major sources of cadmium exposure include jewelry, abandoned electronics, and tobacco smoking [16, 25]. Arsenic is found in cigarette smoke, cosmetics, herbicides, pesticides, and insecticides [16, 26]. Exposure to these heavy metals negatively impact both the growth and development of the fetuses [5, 16, 27]. Also, this exposure disrupts the complex cellular processes that support lung parenchyma regeneration and maintain alveolar epithelium homeostasis [28].

The significant increases in the heavy metals levels in neonates with NRDS and their correlations with the levels of SP-D, CTnI, and hs-CRP goes with the findings of many previous studies. Lead is known to cross the placenta and adversely affects fetuses [16, 29]. Also, cadmium could cross the placenta and negatively impact the pregnancy being a cytotoxic and endocrine disruptor agent. It interferes with placental calcium and nutrients transport, lowering fetal birth weight and causing preterm births [16, 25, 30]. In addition, chronic arsenic poisoning is incredibly harmful to the human lungs [31].

In a study that was conducted by Daston (1981), a disrupted production of the fetus's pulmonary surfactant, decreased lung weight and increased postnatal mortality due to respiratory distress were noticed after giving Cd pregnant rats [32].

Acute Pb poisoning impairs surfactant synthesis and disrupts the lung parenchyma and normal respiratory function [33]. The pretreatment of rats with lead acetate destroyed the lung surfactant, and damaged the surfactant layer and the laminae within the lamellar bodies of the epithelial type II cells [33]. Chen et al. (2014) found that maternal exposure to Pb and Cd causes early fetal exposure through the maternal–fetal transfer of these heavy metals, so that early blood Pb testing is essential for early intervention and clinical decision-making [29]. In addition, Ali et al. (2020) found that there are significant differences in smoking status of newborns mothers when comparing RDS newborns and normal newborns, and also reported that the concentrations of the whole blood Pb was significantly higher in neonates with RDS than normal neonates[34]. Moreover, Wang et al.(2020) who reported that the exposure to As badly affected the pulmonary function especially in smoking subjects [28].

Turker et al. (2013) reported that lead and cadmium levels in meconium were associated with a high percentage of NRDS and neonatal mortality. These levels were found to be higher in non-surviving neonates than in surviving neonates and the incidence of NRDS and intrauterine growth retardation was significantly high in non-surviving neonates [35]. On the other hand, while found significant high levels of Pb, Ali et al. (2020) reported a non-significant differences regarding blood Cd levels when comparing neonates with RDS to normal neonates [34].

In the current study, the high levels of heavy metals in the maternal and neonatal samples in the NRDS group may be partially attributed to the maternal exposure to passive smoking, insecticides, and pesticides which are known sources for these toxins [5].

Surfactant protein D belongs to the collectin group of defense lectins. Because of its surface tension-lowering properties and vital role in innate immunity, it is important for lung health and optimal respiratory function [36]. In early pregnancy, SP-D production is low and grows as the pregnancy progressed but declines again late in pregnancy which explains the low levels of serum SP-D in neonates [37]. SP-D is a likely marker for lung diseases because disease-induced breakdown or changes enhance its systemic leaking from the lungs, so disturbances in SP-D was reported in several neonatal diseases including lung infections, RDS, and bronchopulmonary dysplasia [37–39]. Serum surfactant protein D (SP-D) levels are fair indicator of pulmonary damage. They are related to the severity of NRDS [40].

The present study found significant higher levels of blood SP-D in neonates suffering from NRDS than healthy control and these levels correlated with cord blood Pb, N-As and maternal U-As, and maternal U-Cd. Especially in preterm newborns, high serum SP-D is linked to respiratory diseases including RDS [41]. These increased serum levels are due to the concentration gradient that permits the pulmonary SP-D to leak into the circulation in cases of lung damage [39]. Dahl et al. (2006) found high levels of serum SP-D in the preterm newborns with NRDS and attributed this to the increased placental and other extrapulmonary production [42].

In contrast to our results, Ider et al. (2021) reported lower serum SP-D levels in NRDS than the healthy neonates [43]. Also, Hilgendorf et al. (2005), found lower serum SP-D levels in preterm infants than full term infants [44].

The stress biomarkers such as CTnI, and hs-CRP have been considered as biomarkers for neonatal stress and indicators of perinatal asphyxia [45]. The significant high levels of CTnI and hs-CRP in NRDS and their positive correlations with heavy metals levels emphasize their role in detecting, monitoring, or prognosticating neonatal respiratory distress [46]. High CTnI levels were reported in ventilated infants with idiopathic moderate RDS than healthy neonates, and these levels correlated with the severity of the cases [47, 48]. Measurement of CTnI may be more beneficial marker in the early detection of preterm newborns at risk of myocardial ischemia during RDS [47].

On the contrary to the result of the current study, Sorokin et al. (2014) found non-significant association between serum cord blood CRP and development of NRDS, and also reported that the logistic regression analysis revealed that lower risks of RDS were associated with higher umbilical cord serum CRP concentrations in neonates with 32 GA [49].

Regarding ROC analysis, except for the hs-CRP, none of the studied parameters showed a good AUC, sensitivity, or specificity to differentiate neonates with RDS from healthy control. Unfortunately, we found no similar studies in this regard to compare with them.

The main limitation of the study is being of a small size.

## Conclusions

Trials should be taken toward minimizing exposure to heavy metals sources as heavy metals toxicity may be accused to be one of the causes of NRDS especially if other apparent causes are not there. Also, we recommended to monitor cadmium and arsenic levels in risky cases during pregnancy.

### Limitation of the study

The main limitation of the current study is the inclusion of both preterm and full-term babies in both the control and the NRDS groups and the dependence on the biochemical data only in the determination of the severity of the NRDS. Future research should consider the preterm infants only in both groups and depend on both clinical and laboratory data for determining the severity of the NRDS.

## Data Availability

The datasets used and/or analyzed during the current study are available from the corresponding author on reasonable request.

## Abbreviations

APGAR: Appearance, pulse, grimace, activity, and respiration
As: Arsenic
AUC: Area under receiver operating characteristic curve
Cd: Cadmium
CTnI: Cardiac troponin I
EDTA: Ethylenediamine tetraacetic acid
ELISA: Enzyme-linked immunosorbent assay
E-waste: Electronic waste
GA: Gestation age
hs-CRP: High sensitive C- reactive protein
IRB: Institutional review board
N-As: Neonatal scalp hair arsenic
NRDS: Neonatal respiratory distress syndrome
Pb: Lead
PM: Particulate matter
PS: Pulmonary surfactant
RBC: Red blood cell
RDS: Respiratory distress syndrome
ROC curve: Receiver operating characteristic curve
SP-D: Surfactant protein D
SPs: Surfactant proteins
